# Ultrasound extraction and bioactive flavonoid profiling of *Alsophila spinulosa* leaves across maturity stages

**DOI:** 10.1016/j.ultsonch.2026.107847

**Published:** 2026-04-13

**Authors:** Xiong Huang, Yingying He, Wenjing Miu, Yu Sui, Pengpeng Gong, Ruixue Yuan, Xuelian Tang, Chen Liu

**Affiliations:** a College of Forestry, Forest Ecology and Conservation in the Upper Reaches of the Yangtze River Key Laboratory of Sichuan Province, Sichuan Agricultural University, Chengdu 611130, Sichuan, PR China; b College of Horticulture, Sichuan Agricultural University, Chengdu 611130, Sichuan, PR China

**Keywords:** *Alsophila spinulosa*, Leaf maturity, Ultrasound-assisted extraction, Flavonoids, Anti-inflammatory, Antioxidant

## Abstract

*Alsophila spinulosa* leaves are consumed as an herbal infusion, but the flavonoid-rich products obtainable from leaves at different maturity stages and their major bioactive constituents remain unclear. In this study, flavonoids from *A. spinulosa* leaves at three maturity stages were investigated by combining ultrasound-assisted extraction optimization, purification using AB-8 macroporous adsorption resin, structural characterization, targeted LC-MS/MS profiling, molecular docking, and cell-based validation in lipopolysaccharide (LPS)-stimulated RAW 264.7 murine macrophages. A four-factor Box-Behnken design optimized extraction time, ultrasonic power, liquid-to-solid ratio, and ethanol concentration, and the practical optimum yielded 61.9 ± 1.73 mg rutin equivalents/g dry weight total flavonoids. The enriched extract was characterized by Fourier-transform infrared spectroscopy, X-ray diffraction, and thermogravimetric analysis, indicating a polyhydroxylated aromatic, largely amorphous product that was sensitive to light and strong alkali. Targeted LC-MS/MS annotated 166 flavonoid metabolites and revealed clear maturity-dependent remodeling, mainly involving flavones and flavonols; isoorientin, vitexin, nicotiflorin, and orientin were the most abundant constituents. Molecular docking highlighted sophoricoside and sieboldin as candidate ligands for Kelch-like ECH-associated protein 1 (Keap1) and nuclear factor kappa B (NF-κB). In LPS-stimulated RAW 264.7 macrophages, the purified extract at 50 μg/mL and six representative monomers, each tested at 10 μM, reduced pro-inflammatory transcript levels and modulated cytoprotective defense-related genes. Overall, these results provide a practical basis for maturity-guided utilization and development of bioactive flavonoid products from *A. spinulosa* leaves.

## Graphical abstract

**Figure:**
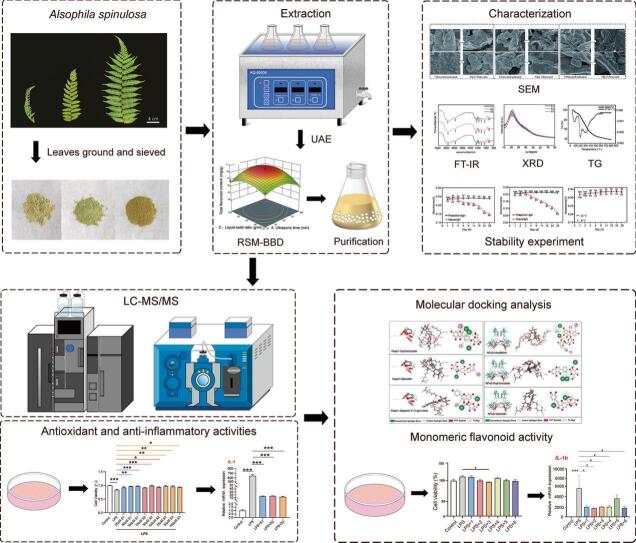

## 1 Introduction

*Alsophila spinulosa* (Cyatheaceae*, genus Alsophila*)*,* a relict lineage of ancient origin, has long attracted attention regarding its phylogeny and taxonomy [1]. In some regions, its leaves have traditionally been infused as a beverage. Modern studies further indicate that *A. spinulosa* is rich in bioactive constituents, and its extracts show antioxidant and anti-inflammatory potential in vitro and in relevant model systems, supporting the development of *A. spinulosa* leaves as a natural source of functional ingredients [2], [3].

Flavonoids are among the major bioactive constituents in plant tissues, and their functional effects often depend more on compositional patterns and key metabolites than on total content alone; therefore, compositionally interpretable profiling is essential for functional evaluation and standardisation [4]. In practice, extract bioactivity often depends on compositional patterns and the contribution of a limited number of indicated molecules rather than total content alone; thus, reproducible preparation together with compositionally interpretable profiling is essential for functional evaluation and standardisation.

Leaf maturity is an important determinant of flavonoid metabolism. As leaves develop from juvenile to mature stages, shifts in light exposure, stress status, and resource allocation can drive stage-dependent remodelling of flavonoid biosynthesis and modification, resulting in changes in subclass composition and the abundance of key metabolites. Targeted LC-MS/MS metabolomics combined with multivariate models has been widely used to resolve developmental-stage-dependent flavonoid variation and identify discriminant metabolites in plants; representative studies are summarised in Supplementary Material 2 (Table S1).

At the process level, total flavonoids are commonly obtained via microwave-assisted extraction, enzyme-assisted extraction, or ultrasound-assisted extraction (UAE) [5]. UAE relies on acoustic cavitation-induced microjets, shear forces, and microstreaming, which enhance solvent penetration and solute release, thereby reducing mass-transfer limitations and improving extraction kinetics under mild conditions [6]. UAE has been validated for leaf flavonoid extraction in systems such as Moringa and basil leaves [7]. However, UAE outcomes are jointly governed by sonication time, acoustic power input, liquid-to-solid ratio, solvent polarity (e.g., ethanol concentration), and their interactions; hence, response surface methodology (RSM) is better suited than one-factor-at-a-time approaches for identifying robust optima. Wang et al. used RSM to model and optimise total flavonoid extraction from perilla leaves and achieved an improved yield [8]. Moreover, coupling RSM-UAE optimisation with targeted LC–MS/MS and OPLS-DA enables simultaneous process optimisation and compositional interpretation, facilitating the screening of key differential metabolites and prioritisation of core molecules [9]. Against this background, a systematic study of *A. spinulosa* leaves is still needed to combine standardised extraction, maturity-dependent flavonoid profiling, and evaluation of the bioactivity of representative flavonoids.

Functionally, the LPS-stimulated RAW 264.7 macrophage model is widely used to assess anti-inflammatory activity and related stress responses. Tracing extract-level effects to specific flavonoid molecules and supporting prioritisation with target-related evidence may improve mechanistic interpretability. NF-kB and Keap1 are two important pathways for anti-inflammation and antioxidant defense. The transcription factor NF-kB is key regulators of inflammatory responses and cells combat ROS through antioxidant defenses, with Keap1 serving as the core regulatory pathway[10], [11]. Molecular docking was employed to evaluate binding affinity and interaction patterns [12]. Structural information for selecting validation candidates can be obtained through molecular docking, based on the roles of NF-kB in inflammation and Keap1 in stress regulation.

Accordingly, this study used *A. spinulosa* leaves at three maturity stages (S1–S3) and applied a Box–Behnken RSM design to optimise UAE conditions using total flavonoid content (TFC) as the response variable. The purified products were characterised by resin purification, FT-IR, XRD, TG, and stability tests under light, temperature, and pH. Targeted LC–MS/MS was performed to profile maturity-dependent flavonoid composition and identify key differential metabolites. The transcriptional responses related to inflammation and cellular defence were evaluated in LPS-stimulated RAW 264.7 cells for purified total flavonoids and prioritised flavonoid monomers, and molecular docking against Keap1 and NF-κB was conducted to assist candidate selection.

Against this background, a framework for *A. spinulosa* leaves that links extraction optimisation, maturity-dependent flavonoid profiling, and bioactivity evaluation remains to be established. In particular, it is still unclear how leaf maturity influences flavonoid composition, which flavonoids are enriched at different developmental stages, and which representative constituents may contribute most to the observed anti-inflammatory potential. Therefore, the aim of this study was to optimise the ultrasound-assisted extraction of flavonoids from *A. spinulosa* leaves, characterise maturity-dependent differences in flavonoid composition by targeted LC-MS/MS, and evaluate the anti-inflammatory potential of the purified flavonoid fraction and representative candidate monomers.

## 2 Methods

### 2.1 Materials and chemicals

**Fig. 1:**
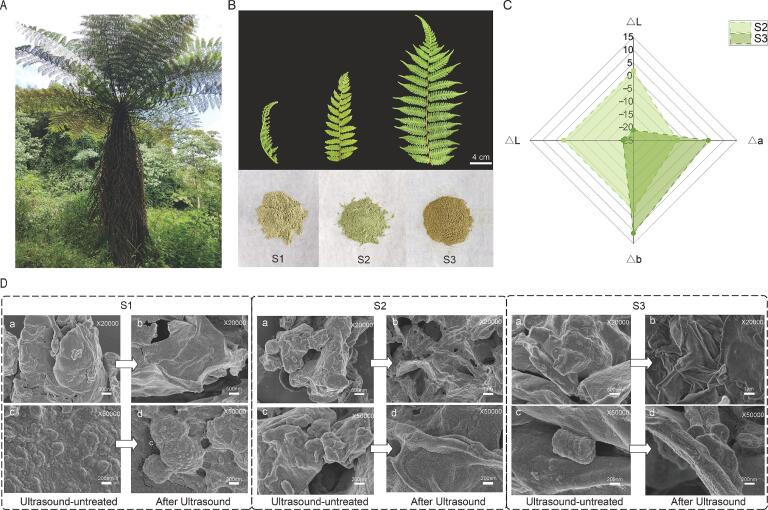
Morphology, colour traits, and SEM microstructure of *A. spinulosa* leaves at three maturity stages (S1-S3) before and after ultrasound treatment. (A) Representative plant image. (B) Representative fronds (top) and corresponding dried powders (bottom) from S1-S3. Scale bar = 4 cm. (C) Powder colour differences of S2 and S3 relative to S1, expressed as ΔL*, Δa*, and Δb*. (D) SEM images of S1-S3 powders before and after ultrasound treatment at × 20,000 and × 50,000 magnification.

Leaf samples of *A. spinulosa* were collected from the National Germplasm Resources Centre, Hongya County, Sichuan Province, China (29.90°N, 103.14°E). Three healthy 10-year-old plants with comparable growth status were selected as biological replicates. Fresh leaves were harvested and categorised into three maturity grades (S1–S3), with ∼3 kg collected per grade. S1 (early developmental stage): leaves collected from the apical position, not fully expanded, with a curled rachis and relatively small laminae; S2 (mid-developmental stage): leaves collected from the middle canopy position, with fronds gradually unfolding and pinnae morphology becoming clearly distinguishable; S3 (mature stage): leaves collected from the basal position, with fully expanded fronds and a darker green colour, indicating a more advanced stage of maturity (Fig. 1B). After transport to the laboratory, samples were air-dried in a well-ventilated shaded area to constant weight, ground, and passed through a 60-mesh sieve. The resulting powders were sealed in airtight bags and stored under dry and light-protected conditions until use.

Ethanol (analytical grade or higher) was used as the extraction solvent. For targeted flavonoid metabolomics, acetonitrile, methanol, and formic acid were of HPLC grade (or LC–MS grade), and ultrapure water was produced using a purification system. Aluminium chloride, sodium nitrite, and sodium hydroxide used for total flavonoid content (TFC) determination were of analytical grade or higher. The murine macrophage cell line RAW 264.7 (CL-0190) was obtained from Wuhan Pricella Biotechnology Co., Ltd. (Wuhan, China). Cell culture reagents including DMEM (high glucose, C11995500BT), fetal bovine serum (FBS, A5669701), and penicillin–streptomycin (PS) solution were cell-culture grade and purchased from Gibco (UK). Lipopolysaccharide (LPS; L2880) used to induce the inflammatory model was purchased from Sigma-Aldrich (USA). The Cell Counting Kit-8 reagent (CCK-8; C0037) was obtained from Beyotime (China). TRIzol reagent for RNA extraction (15596026CN), as well as reverse transcription and real-time qPCR kits (SYBR Green chemistry), were purchased from Vazyme (China). Six flavonoid reference standards intended for molecular docking-based prioritisation and subsequent bioactivity validation—vitexin (98.0%, PUSH), isoorientin (98.5%, PUSH), orientin (98.5%, PUSH), nicotiflorin (98.5%, PUSH), sophoricoside (99.0%, PUSH), and sieboldin (98.0%, Purify)—were chromatographic-grade standards. Unless otherwise stated, all other chemicals were of analytical grade or higher.

### 2.2 Experimental design of ultrasound-assisted extraction (UAE)

#### 2.2.1 Single-factor experiments

To identify key process variables for ultrasound-assisted extraction (UAE) and to define suitable ranges for subsequent response-surface optimisation, single-factor experiments were conducted using *A. spinulosa* leaf powder as the raw material. Total flavonoid content (TFC, mg/g) was used as the response variable. While keeping the remaining conditions constant (including sample mass, total solvent volume, the ultrasound apparatus, and the operating mode), the effects of sonication time (A), ultrasonic power (B), liquid-to-solid ratio (C), and ethanol volume fraction (D) on TFC were evaluated.

Briefly, preliminary screening was first performed across broad parameter ranges to capture the overall response trends. Based on these results, the factor ranges used for the Box–Behnken design (BBD) were set as follows (Table S1 in Supplementary material1): sonication time 20–100 min (centre point, 60 min), ultrasonic power 100–500 W (centre point, 300 W), liquid-to-solid ratio 20–100 (g/mL) (centre point, 60 (g/mL)), and ethanol volume fraction 20–80% (centre point, 50%).

#### 2.2.2 Response surface design and statistical analysis (BBD-RSM)

On the basis of the single-factor experiments, ultrasonic time (A, min), ultrasonic power (B, W), liquid-to-solid ratio (C, g/mL), and ethanol volume fraction (D, %) were selected as independent variables, with TFC (mg/g) as the response. A four-factor, three-level Box–Behnken design (BBD) was employed to optimise the UAE conditions for flavonoid extraction from *A. spinulosa* leaves. The actual and coded levels (−1, 0, +1) of each factor are shown in Supplementary material 1 (Table S2): A = 20, 60, 100 min; B = 100, 300, 500 W; C = 20, 60, 100 g/mL; and D = 20, 50, 80%.

Experimental design and data analysis were performed using Design-Expert software. A four-factor, three-level Box-Behnken design with 29 runs, including five centre points, was used to optimise the extraction conditions. The experimental results were fitted to a second-order polynomial model, and analysis of variance (ANOVA) was used to assess the significance and adequacy of the model. Response surface and contour plots were generated to evaluate the effects of the independent variables and their interactions on the response. Numerical optimisation was subsequently carried out to predict the optimal extraction conditions. The model can be expressed as:

(1) $$\mathrm{TFC}=\beta_{0}+\beta_{A}A+\beta_{B}B+\beta_{C}C+\beta_{D}D+\beta_{\mathrm{AB}}AB+\beta_{\mathrm{AC}}AC+\beta_{\mathrm{AD}}AD+\beta_{\mathrm{BC}}BC+\beta_{\mathrm{BD}}BD+\beta_{\mathrm{CD}}CD+\beta AAA^{2}+\beta BBB^{2}+\beta CCC^{2}+\beta DDD^{2}$$

where TFC is the total flavonoid content (mg/g); A–D are the coded independent variables; *β*_0_ is the intercept; *β*_A_-*β_D_* are the linear coefficients; *β_AB_* are the interaction coefficients; and *β_AA_* are the quadratic coefficients.

The significance of the regression model and model terms was assessed by analysis of variance (ANOVA), with P < 0.05 considered statistically significant. Model adequacy was evaluated using the coefficient of determination (R^2^), adjusted R^2^ (Adj-R^2^), lack-of-fit test, and residual diagnostics. Three-dimensional response surface and contour plots were generated to visualise factor interactions and to identify optimal UAE conditions. Finally, validation experiments were performed under the predicted optimum conditions (n = 3), and the experimental TFC was compared with the model-predicted value to calculate the relative error and assess the predictive performance and process robustness.

### 2.3 Purification

AB-8 is widely used for flavonoid purification. Owing to its weak polarity, it can effectively remove and enrich pigments, proteins, sugars, and other highly polar co-extractives, and it offers high selectivity, stability, and efficiency [13]. Therefore, in this study, AB-8 was used to enrich the total flavonoids from *A. spinulosa* leaves and improve sample purity. Prior to use, the resin was soaked in 95% (v/v) ethanol for 4 h to remove impurities and then thoroughly rinsed with ultrapure water until no ethanol odour remained. The UAE extracts were filtered to remove insoluble residues. Ethanol was removed under reduced pressure, and the concentrates were reconstituted to a defined volume with ultrapure water to obtain the loading solutions.

For each maturity grade (S1-S3), the corresponding loading solution was mixed with an equal amount of pretreated AB-8 resin in a stoppered vessel and incubated in the dark on a temperature-controlled orbital shaker at 20 °C for 12–14 h to allow adsorption, according to Shu et al. [14], with minor modifications. After adsorption, the supernatant was discarded and the resin was rinsed with ultrapure water to remove weakly adsorbed impurities. Flavonoids retained on the resin were then eluted with 60% (v/v) ethanol, and the eluates were collected. The eluates were concentrated by rotary evaporation under reduced pressure and subsequently freeze-dried under vacuum to obtain purified flavonoid powders, which were sealed and stored protected from light until further analysis. The detailed experimental flowchart is provided in Supplementary Material 1 (Fig. S1).

### 2.4 Determination of total flavonoid content (TFC)

The total flavonoid content (TFC) of *A. spinulosa* leaf extracts was determined by the AlCl_3_ spectrophotometric method according to [15]. Rutin was used as the reference standard to prepare a series of standard solutions. After colour development under the same conditions as the samples, the absorbance of each standard solution was measured and a calibration curve was constructed by plotting absorbance (y) against rutin concentration (x). Sample solutions were subjected to the same colour development procedure, and absorbance was measured at the designated wavelength. The flavonoid concentration in the sample solution was calculated from the calibration curve as (C) (mg/mL) and then converted to a dry-weight basis.

TFC was calculated using the following equation:

(2) $$\text{TFC}\left(\text{mg}\;\text{RE}/\text{g}\;\text{DW}\right)=\frac{\text{C}\times \text{V}\times \text{DF}}{\text{m}}$$

where (C) is the flavonoid concentration of the sample solution derived from the calibration curve (mg/mL); (V) is the total volume of the sample solution (mL); (DF) is the dilution factor prior to measurement (DF = 1 if undiluted); and (m) is the mass of dry *A. spinulosa* leaf powder used for extraction (g). Results are reported as mg rutin equivalents per g dry weight (mg RE/g DW).

### 2.5 Scanning electron microscopy (SEM)

Scanning electron microscopy (SEM) was used to compare the surface microstructures of *A. spinulosa* leaf samples at different maturity grades (S1–S3) before and after ultrasound-assisted extraction (UAE). Samples collected prior to UAE were air-dried leaf powders. Post-UAE samples were the solid residues recovered at the end of extraction (filtration cake and/or centrifugation pellet). The residues were briefly rinsed with ultrapure water to remove residual solvent and then dried to constant weight by oven drying (or vacuum drying).

A small amount of each dried sample was evenly distributed on aluminium stubs, fixed with conductive adhesive, and sputter-coated with gold to improve conductivity. Micrographs were acquired at an accelerating voltage of 3.0 kV and a working distance of 5.0 mm, with magnifications of × 20,000 and × 50,000. For each sample, images were captured from multiple random fields of view to ensure representative morphology.

### 2.6 Fourier-transform infrared spectroscopy (FT-IR)

Fourier-transform infrared spectroscopy (FT-IR) was performed to characterise the functional-group features of the total flavonoid powders. Samples were prepared using the KBr pellet method. Briefly, 1.0 mg of dried total flavonoid powder was thoroughly mixed with 50.0 mg of dry KBr in an agate mortar under dry conditions, and the mixture was pressed into a homogeneous transparent pellet. A blank KBr pellet was used as the background. Spectra were recorded over 4000–400 cm^−1^ at a resolution of 4 cm^−1^ with 150 accumulated scans.

### 2.7 Thermogravimetric analysis (TGA)

Thermogravimetric analysis (TGA) was conducted to evaluate the thermal behaviour of the total flavonoid powders using a METTLER TOLEDO thermogravimetric analyser under a nitrogen atmosphere. Approximately 5–10 mg of sample was placed in an alumina crucible and heated from 40 °C to the specified final temperature according to the instrument programme. The mass-loss curve (TG) and its derivative (DTG) were recorded as a function of temperature.

### 2.8 X-ray diffraction (XRD)

Powder X-ray diffraction (XRD) was used to assess the crystallographic characteristics of the total flavonoid powders. An appropriate amount of powder was placed on a single-crystal silicon sample holder and gently levelled with a spatula (or weighing paper) to obtain a flat and compact surface, thereby minimising preferred orientation and surface-roughness effects. XRD patterns were collected using Cu Kα radiation (λ = 1.5418 Å) at 40 kV and 40 mA. Data were acquired in continuous scan mode over 2θ = 5–90° with a counting time of 0.1 s per step. The diffractograms were used to evaluate crystallinity and the presence/absence of characteristic diffraction peaks.

### 2.9 Stability experiment of total flavonoid content (TFC)

“To evaluate the stability of total flavonoids in *A. spinulosa* leaf extracts in solution, the effects of storage temperature, pH, and light exposure on the retention rate of total flavonoid content (TFC) were investigated according to a reference method [16]. Briefly, TFC solutions with the same initial concentration (denoted as C_0_) were prepared using the same solvent system and preparation procedure, and then aliquoted into sealed containers for different treatments. At each sampling time point, TFC was determined according to the method described in Section 2.4. This experiment was conducted to provide basic information on the storage stability, handling requirements, and potential shelf-life of the developed flavonoid extract.

TFC retention was calculated using the initial value measured at Day 0 (TFC_0_) as the reference:

(3) $$\text{Retention}\left(\%\right)=\frac{\text{TFCt}}{\text{TFC}0}\times 100$$

where TFC_t_ is the measured TFC value under a given condition at time *t*.

#### 2.9.1 Photostability

Aliquoted TFC solutions were stored in sealed containers under natural light. Sealed light-protected samples prepared from the same batch (identical container type and ambient temperature) were used as controls. Sampling was performed once daily at 16:00 during the first 4 days, followed by sampling every 5 days over the subsequent 25 days. After each sampling, TFC was immediately determined according to Section 2.4.

#### 2.9.2 Thermal stability

Aliquoted TFC solutions were stored in sealed containers at 4 °C and 25 °C, respectively. For each temperature, sealed light-protected samples were included as controls. The sampling schedule was identical to that described above (Section 2.9.1): once daily at 16:00 during the first 4 days, and then every 5 days for the following 25 days. TFC was quantified after sampling as described in Section 2.4.

#### 2.9.3 pH stability

TFC solutions were adjusted to pH 1, 3, 5, 7, 9, 11, and 13 using acid/base solutions. The pH meter was calibrated prior to measurements. After standing for 1 h in the dark, TFC was determined according to Section 2.4. The solution volume and initial flavonoid concentration were kept consistent before and after pH adjustment to minimise dilution-related bias.

### 2.10 Targeted flavonoid profiling by LC–MS/MS

To systematically characterise the flavonoid spectrum of *A. spinulosa* leaves, a targeted metabolomics workflow was applied based on an in-house metabolite database covering 345 plant flavonoids. The method combined external calibration with (where applicable) isotope-labelled internal standards. Representative flavonoid reference standards, including quercetin, were purchased from MCE (Monmouth Junction, NJ, USA) and prepared as 10 mM stock solutions in 70% (v/v) methanol.

For target flavonoids with available authentic standards, compound identities were confirmed by matching retention times and MS/MS fragment-ion patterns, and absolute quantification was performed using external calibration. For targets without available standards, identification was achieved based on predefined MRM transitions together with characteristic product-ion information, and relative quantification was performed accordingly.

#### 2.10.1 Sample preparation

An appropriate amount of total flavonoid powder was mixed with 70% (v/v) methanol and extracted by ultrasonication for 30 min. The mixture was centrifuged at 4 °C (12,000 rpm, 5 min), and the supernatant was collected, passed through a 0.22 μm membrane filter, and transferred to autosampler vials for LC–MS/MS analysis.

#### 2.10.2 UPLC conditions

Chromatographic separation was carried out on an ExionLC™ AD UHPLC system (SCIEX, USA) equipped with a Waters ACQUITY UPLC HSS T3 C18 column (1.8 μm, 100 mm × 2.1 mm i.d.). Mobile phase A was ultrapure water containing 0.05% (v/v) formic acid, and mobile phase B was acetonitrile containing 0.05% (v/v) formic acid. The flow rate was set at 0.35 mL/min, the column temperature at 40 °C, and the injection volume at 2 μL. The gradient programme (A/B, v/v) was: 0 min, 90:10; 1 min, 80:20; 9 min, 30:70; 12.5 min, 5:95; 13.5 min, 5:95; 13.6 min, 90:10; and 16 min, 90:10.

#### 2.10.3 MS/MS conditions and quantification strategy

MS/MS detection was performed on a QTRAP® 6500 + mass spectrometer (SCIEX, USA) equipped with an electrospray ionisation (ESI) source, operating in multiple reaction monitoring (MRM) mode. The source temperature was 550 °C. The ion spray voltage was set to 5500 V in positive-ion mode and − 4500 V in negative-ion mode. Curtain gas (CUR) was maintained at 35 psi. Other source and compound-dependent parameters were set according to the manufacturer’s recommendations and further optimised based on the target-specific MRM transitions.

External calibration curves were constructed for quantification, and acceptable linearity was defined as a correlation coefficient (R) ≥ 0.9900. Details of calibration curves are provided in Supplementary material 2 (Table S2).

### 2.11 Cell culture and treatments

The murine macrophage cell line RAW 264.7 was cultured in Dulbecco’s modified Eagle’s medium (DMEM) supplemented with 10% (v/v) heat-inactivated fetal bovine serum (FBS) and 1% (v/v) penicillin–streptomycin at 37 °C in a humidified incubator with 5% CO_2_. Cells were routinely passaged and used at ∼80% confluence.

For inflammatory stimulation, cells were exposed to lipopolysaccharide (LPS, 200 ng/mL) in the absence or presence of purified flavonoid extracts (S1–S3) or individual flavonoid monomers at the indicated concentrations for 24 h. Where applicable, the experimental design included an untreated control, an LPS-only control, and extract/monomer-only controls.

### 2.12 Cell viability assay

Cell viability was evaluated using a Cell Counting Kit-8 (CCK-8). Following 24 h treatment with flavonoid extracts or individual flavonoid monomers, 10 μL of CCK-8 reagent was added to each well and plates were incubated for 1 h at 37 °C. Absorbance at 450 nm was recorded using a SpectraMax 190 microplate reader. After background subtraction, cell viability was expressed as a percentage of the untreated control.

### 2.13 RT–qPCR analysis

Total RNA was extracted using RNA Isolater Total RNA Extraction Reagent following the manufacturer’s instructions. First-strand cDNA was synthesised with HiScript II Q RT Supermix. Quantitative PCR was carried out using ChamQ SYBR Color qPCR Master Mix on a 7900HT Real-Time PCR System (Applied Biosystems).

The thermal cycling programme comprised an initial denaturation at 95 °C for 1 min, followed by 35 amplification cycles of 95 °C for 15 s and 60 °C for 30 s. Melt-curve analysis (95 °C for 15 s, 60 °C for 15 s, and 90 °C for 15 s) was performed to verify amplification specificity. Relative mRNA levels were calculated using the 2^−ΔΔCt method and normalised to GAPDH. Primer sequences are listed in Supplementary material 1 (Table S3). Unless otherwise stated, experiments were performed with at least three independent biological replicates.

### 2.14 Molecular docking analysis

The three-dimensional structures of Keap1 and NF-κB were retrieved from the UniProt database. Ligand structures (e.g., vitexin, luteolin-6-C-glucoside) were downloaded from PubChem and prepared according to the docking server requirements. Molecular docking was carried out using the CB-Dock2 online server under default parameters. The top-ranked poses were prioritised based on the predicted binding affinity and cavity score. Protein–ligand binding conformations and key interactions, including hydrogen bonds, hydrophobic contacts, and π-interactions, were visualised and analysed using Discovery Studio 2019 (Dassault Systèmes BIOVIA, San Diego, CA, USA).

### 2.15 Statistical analysis

Data processing and visualisation were performed using Origin 2024 (OriginLab, Northampton, MA, USA) and GraphPad Prism (GraphPad Software, San Diego, CA, USA). Response surface methodology (RSM) modelling and response surface/contour plot generation were conducted using Design-Expert v13.0.0 (Stat-Ease Inc., Minneapolis, MN, USA). Targeted flavonoid metabolomics data were processed and analysed using Metware Cloud (Metware Biotechnology Co., Ltd., Wuhan, China). All data are presented as mean ± standard deviation (SD) (n = 3). Statistical significance was evaluated using appropriate parametric tests (as specified in the figure legends), and P < 0.05 was considered statistically significant.

## 3 Results and discussion

### 3.1 Phenotypic variation across leaf maturity stages and microstructural responses to ultrasound treatment

As shown in Fig. 1A–B, A*. spinulosa* leaves displayed a clear maturity gradient from S1 to S3. The corresponding dried powders (Fig. 1B, lower row) also differed markedly in appearance, suggesting that maturation is accompanied not only by morphological transition but also by systematic remodelling of pigments and phenolic constituents. This provides an intuitive phenotypic basis for the subsequent association analysis between maturity stage, flavonoid profiles, and bioactivity.

To quantify maturity-associated colour variation, colour difference analysis was performed using the S1 powder as the reference (Fig. 1C). The results showed an increase in the lightness parameter (ΔL) in S2, consistent with a visually brighter powder. With further maturation to S3, ΔL continued to increase, while both Δa and Δb shifted substantially, indicating an overall tendency towards a more yellowish tone. Such colour changes are commonly observed during leaf maturation and senescence and are often related to a relative decline in chlorophyll contribution together with increased relative contributions from carotenoids and oxidised phenolics [17]. In this context, the colourimetric shift in Fig. 1C can be considered a macroscopic readout of underlying chemical reconfiguration, which is subsequently examined at the metabolite level by targeted flavonoid profiling.

SEM micrographs of powders before and after ultrasound treatment are presented in Fig. 1D. Prior to sonication, powders from all three maturity stages appeared as densely aggregated particles with a relatively continuous surface, abundant wrinkles, and limited visible porosity. Localised granular deposits were observed on the particle surfaces, which may reflect dried cellular contents or aggregated polyphenolic components formed during dehydration. After ultrasound treatment, all samples showed pronounced microstructural disruption, characterised by surface erosion, the emergence of fissures and pores, increased lamellar features, and occasional collapse or fracture. These changes indicate an effective weakening of structural barriers, which is expected to facilitate solvent penetration and outward diffusion of soluble constituents.

This microstructural response is consistent with the physical basis of ultrasound-assisted extraction, where acoustic cavitation generates microjets, shockwaves, and strong shear fields that can erode surfaces and disrupt cell-wall and fibre frameworks, thereby reducing interfacial mass-transfer resistance and accelerating target release [18]. This analysis was performed to visually confirm the structural disruption of leaf tissues induced by ultrasonic cavitation and to relate these morphological changes to enhanced solvent penetration and flavonoid extraction efficiency. Notably, differences in tissue compactness and cell-wall maturity across S1–S3 may alter susceptibility to cavitation-induced damage, which could contribute to stage-dependent variations in extraction yield and flavonoid composition observed later.

### 3.2 Optimisation of the UAE process for flavonoids in *a. Spinulosa* leaves using a Box–Behnken response surface model

Based on the single-factor screening (Supplementary material 1, Fig. S2), sonication time (A), ultrasonic power (B), liquid-to-solid ratio (C) and ethanol concentration (D) were selected as key variables for process optimisation (Supplementary material 1, Table S4). A Box–Behnken design (BBD) was then employed to construct a four-factor, three-level response surface model using total flavonoid content (TFC, mg/g) as the response. BBD is a widely used three-level quadratic design that enables efficient estimation of linear, interaction and quadratic effects with a reduced number of experimental runs, making it particularly suitable for UAE processes where multiple parameters are coupled [19].

Using the experimental data (Supplementary material 2, Table S3), the following second-order polynomial regression equation was obtained (coded variables):

$$\text{TFC}=61.83+0.8222\text{A}+1.54\text{B}+2.03\text{C}-3.05\text{D}-1.92\text{AB}+1.86\text{AC}+0.00000\text{AD}+0.1917\text{BC}+0.5250\text{BD}-2.33\text{CD}-4.13{\text{A}}^{2}-0.3189{\text{B}}^{2}-4.11{\text{C}}^{2}-11.04{\text{D}}^{2}.$$

ANOVA results (Supplementary material 1, Table S5) indicated that the model was highly significant (P < 0.0001), while the lack-of-fit was not significant (P = 0.102 > 0.05), supporting good model adequacy within the studied region. The model showed a high coefficient of determination (R^2^ = 0.9660) and adjusted R^2^ (Adj-R^2^ = 0.9320), suggesting that the majority of the variability in TFC could be explained by the fitted equation, with satisfactory robustness and predictive performance.

Regarding main effects, ethanol concentration (D) exhibited a highly significant linear effect on TFC (P < 0.0001). Moreover, the quadratic term D^2^ produced the largest F-value (279.18), indicating a pronounced curvature, consistent with a clear optimum ethanol window rather than a monotonic relationship. This behaviour reflects the dual role of solvent polarity and matrix hydration in flavonoid extraction. Water promotes tissue swelling and solvent penetration, whereas excessive ethanol dehydrates the matrix and restricts diffusion; thus, aqueous ethanol typically outperforms pure solvents and shows an optimum concentration range.

The liquid-to-solid ratio (C) also significantly affected TFC (P = 0.0009), and its quadratic term C^2^ was highly significant (P < 0.0001), implying a non-linear response where the benefit of increasing solvent volume diminishes beyond a certain point. This trend can be attributed to the fact that increasing solvent availability enhances the concentration gradient and dissolution driving force at lower ratios, whereas further increases approach solute–solvent equilibrium and yield marginal gains, a saturation or plateau effect commonly observed in flavonoid extraction from plant matrices [20]. Ultrasonic power (B) showed a significant linear effect (P = 0.0069), reflecting that within the tested range, increasing acoustic energy input improved extraction efficiency, plausibly through stronger cavitation that enhances cell disruption, solvent penetration and boundary-layer renewal, thereby reducing mass-transfer resistance [21]. By contrast, the linear effect of sonication time (A) was not significant (P = 0.1127), whereas A^2^ was highly significant (P < 0.0001), suggesting that time mainly influenced TFC in a non-linear manner, where additional sonication beyond an effective window yields diminishing returns and may not further increase recovery.

**Fig. 2:**
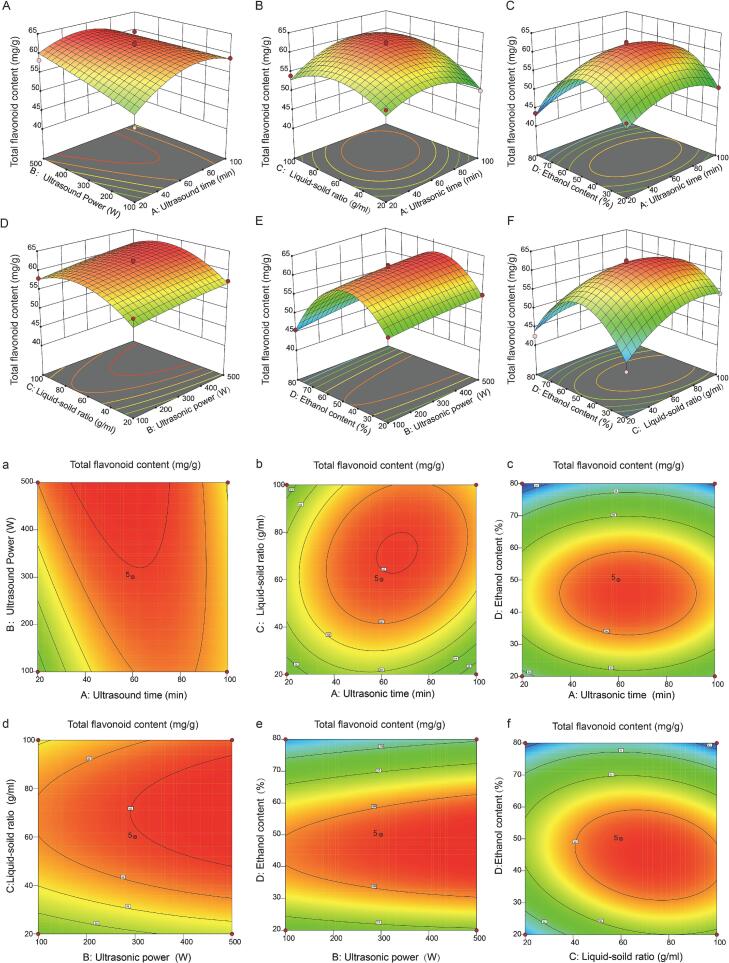
Response surface and contour plots showing the interactive effects of UAE variables on total flavonoid content in *A. spinulosa* leaves. (A, a) Ultrasound time and ultrasound power. (B, b) Liquid-to-solid ratio and ultrasound time. (C, c) Ethanol concentration and liquid-to-solid ratio. (D, d) Liquid-to-solid ratio and ultrasound power. (E, e) Ethanol concentration and ultrasound power. (F, f) Ethanol concentration and liquid-to-solid ratio.

In terms of interactions, AB and AC were significant (P < 0.05), indicating that the effect of sonication time depended on ultrasonic power and liquid-to-solid ratio, consistent with the coupled roles of energy delivery and mass-transfer driving force. CD was also significant (P = 0.0152), implying a combined influence of solvent composition and liquid-to-solid ratio on flavonoid release, likely reflecting their joint effects on solubility, matrix swelling and diffusion gradients. Importantly, the AD term was not significant (P = 1), and therefore the response surface interpretation in Fig. 2 should focus primarily on AB, AC and CD rather than an ethanol concentration by time interaction.

Based on the fitted model and the response surface analysis (Fig. 2), Design-Expert predicted the optimal conditions as: ethanol concentration 44.864%, liquid-to-solid ratio 83.043 (g/mL), sonication time 60.431 min, and ultrasonic power 305.579 W, with a predicted TFC of 62.123 mg/g. Considering practical operability, these conditions were adjusted to 45% ethanol, 300 W, a liquid-to-solid ratio of 1:83 (g/mL), and 60 min for validation. The experimentally measured TFC was 61.9 ± 1.73 mg/g, in good agreement with the predicted value, confirming that the optimised UAE protocol is feasible and reproducible, and can serve as a reliable basis for subsequent purification and downstream analyses. It should be noted that an elevated total flavonoid content does not necessarily correspond to enhanced biological activity or to the maximal recovery of specific bioactive constituents. This reflects a common limitation in natural product extraction optimization, where response surface methodology (RSM) is frequently based on total content assays, such as the AlCl_3_ method for TFC or the Folin-Ciocalteu method for total phenolics, as the sole response variable, without independent validation of key labeled compounds or bioactive constituents ([15], [22]. Future studies should move toward multi-response optimization by incorporating total content, key monomeric flavonoids, and bioactivity-related indicators into the experimental design, so as to establish an extraction process that more accurately reflects the bioactive basis of the material.”.

### 3.3 FT-IR analysis

**Fig. 3:**
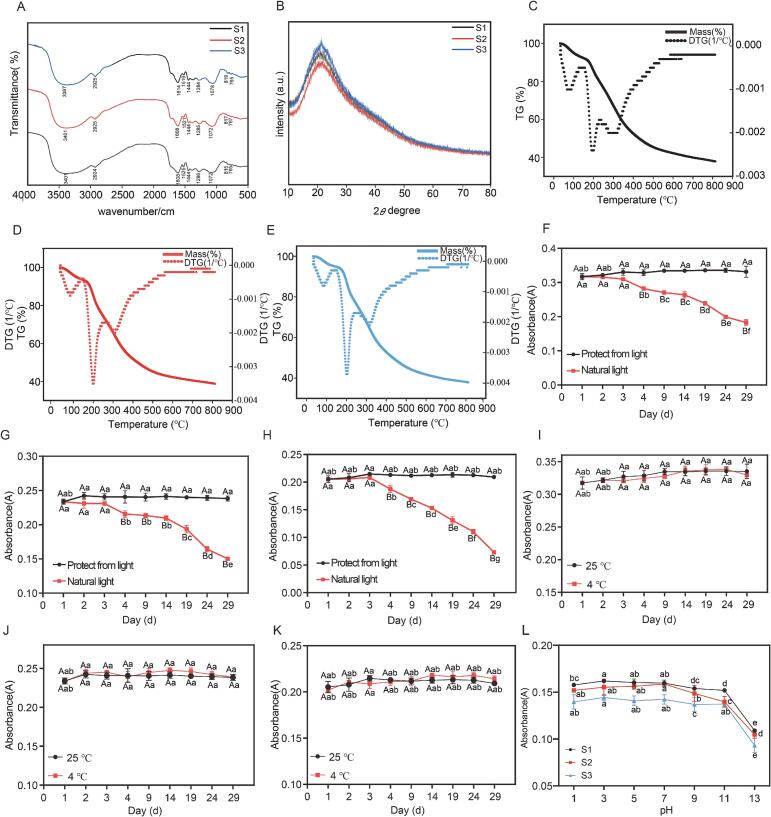
Physicochemical characterization and storage stability of total flavonoid extracts from *A. spinulosa* leaves at three maturity stages (S1-S3). (A) FT-IR spectra. (B) XRD patterns. (C-E) TG and DTG curves of extracts from S1, S2, and S3, respectively. (F-H) Photostability of flavonoid solutions from S1, S2, and S3 under light and dark storage. (I-K) Temperature stability of flavonoid solutions from S1, S2, and S3 at 4 °C and 25 °C under dark conditions. (L) pH stability of flavonoid solutions after 1 h incubation at pH 1–13. Data are presented as mean ± SD (n = 3).

Fourier-transform infrared spectroscopy (FT-IR) is a widely used technique for identifying functional-group signatures and probing hydrogen-bonding interactions in complex matrices [23]. Accordingly, FT-IR spectra were acquired for the total flavonoid powders obtained from *A. spinulosa* leaves across three maturity grades (S1–S3) (Fig. 3A). Overall, the three samples exhibited highly consistent band positions, with only moderate differences in band intensities. This suggests that, at the level of the core structural motifs, the flavonoid-rich fractions were broadly comparable across maturity stages, whereas variations may exist in relative abundances, glycosylation patterns, and/or co-extracted minor constituents. Such intensity-level differences provide a useful spectroscopic context for the subsequent chemical stratification based on targeted metabolomics [24].

Specifically, all three samples showed a broad and intense band centred around ∼3400 cm^−1^, which can be attributed to O–H stretching vibrations of phenolic hydroxyl groups. This feature is characteristic of polyhydroxylated flavonoids and implies the presence of extensive intra- and/or intermolecular hydrogen-bonding networks [25]. A set of relatively sharp bands in the ∼1600 cm^−1^ and ∼1400 cm^−1^ regions is typically associated with aromatic C=C skeletal stretching, consistent with the aromatic backbone of flavonoid structures. In the fingerprint region (approximately 1200–1000 cm^−1^), prominent absorptions corresponding to C–O and C–O–C vibrations were observed, which may arise from phenolic ether linkages and/or glycosidic bonds, further supporting the presence of flavonoids and their glycosylated derivatives in the extracts [26]. In addition, bands around ∼800–820 cm^−1^ are commonly assigned to out-of-plane C–H bending vibrations of substituted aromatic rings, which can reflect differences in aromatic substitution patterns among flavonoid subclasses and substitution types.

It should be noted that FT-IR provides strong indicative evidence at the functional-group level, but it cannot precisely resolve individual constituents in complex mixtures. Therefore, in this study FT-IR serves as a structural “fingerprint” validation tool that complements the targeted LC–MS/MS profiling, together supporting interpretation of compositional variation across maturity stages without over-extending FT-IR beyond its analytical scope.

### 3.4 XRD analysis

X-ray diffraction (XRD) analysis was conducted to characterize the crystalline properties of the flavonoid fraction and assess the physical stability of the solid-state formulation [27]. As shown in Fig. 3B, all three total flavonoid powders exhibited a broad diffuse halo in the 2θ range of ∼16–25°, with no sharp Bragg reflections, indicating that the samples were predominantly amorphous and lacked long-range crystalline order. This pattern is consistent with the well-established criterion that crystalline solids generate distinct diffraction peaks, whereas amorphous materials typically display broad humps/halos due to short-range ordering only [28].

From a processing perspective, resin enrichment (AB-8), solvent removal by rotary evaporation, and subsequent drying can hinder regular molecular packing in the solid state, particularly for multi-component flavonoid systems, thereby favouring the formation of amorphous powders. Amorphous solids generally possess higher free energy and weaker lattice constraints, which may translate into faster dissolution and higher apparent solubility in aqueous media. This feature can be advantageous for downstream bioactivity evaluation, especially in cell-exposure systems where achieving an effective concentration is critical. Meanwhile, amorphous materials are often more hygroscopic and may undergo structural rearrangement or local crystallisation under certain conditions. Therefore, moisture control and protection from light should be prioritised during storage and any subsequent formulation development, and the practical storage strategy can be informed by the TG and stability results presented in this study.

### 3.5 TG analysis

Thermogravimetric (TG) analysis was performed to determine thermal decomposition behaviour, moisture/solvent residues, and to predict storage conditions and upper temperature limits for processing [29]. Fig. 3C–E show the thermogravimetric (TG) and derivative TG (DTG) curves of total flavonoid powders obtained from *A. spinulosa* leaves at three maturity grades (S1–S3). Overall, all samples exhibited the characteristic thermal pattern of an initial minor mass loss followed by a pronounced decomposition stage, indicating that loosely bound components (e.g., adsorbed/bound water and trace volatiles) were released at the early stage, whereas the major mass-loss event at higher temperatures was dominated by thermal breakdown of the flavonoid-enriched matrix and co-existing constituents. Notably, the distribution of mass-loss stages and the positions/shapes of DTG maxima differed among S1–S3, suggesting maturity-dependent variations in compositional features (e.g., glycosylation extent and co-extracted components) and/or the strength of intermolecular interactions, which collectively modulate the apparent thermal stability.

For S1, the TG/DTG profiles displayed a more clearly staged mass-loss behaviour. An initial mass decrease of ∼7% was observed, plausibly attributable to moisture removal (adsorbed/bound water), potentially accompanied by release of trace low-molecular-weight residues. The subsequent major DTG peak corresponded to rapid decomposition of the flavonoid-rich solid. This low-level mass loss (∼7–8 wt%) attributable to water release has also been reported for typical flavonoids such as quercetin hydrate, providing a relevant benchmark supporting the interpretation of the early-stage loss in the S1 sample [29]. From an application perspective, this implies that the S1 powder may be more sensitive to ambient humidity, which could affect powder handling properties (e.g., flowability) and potentially contribute to batch-to-batch variability; thus, sealed and moisture-protective storage is recommended.

In contrast, S2 and S3 exhibited more “concentrated” thermal events, with a single sharp DTG peak occurring at approximately 201.15 °C and 201.07 °C, respectively, and an overall shift of the characteristic temperature towards lower values compared with S1, implying reduced apparent thermal stability. This difference may reflect maturity-associated changes in the flavonoid profile (e.g., altered subclass distribution, glycosylation patterns, or shifts in co-extracted phenolics), which can modify hydrogen-bonding networks and thermal degradation kinetics. In addition, microstructural differences arising from extraction and drying (e.g., porosity, specific surface area, and aggregation state) may alter heat transfer and volatilisation/decomposition behaviour, contributing to peak sharpening or peak-temperature shifts in TG/DTG curves [30]. Importantly, as “total flavonoid powders” represent multicomponent systems, TG/DTG signals should be interpreted as integrated thermal stability windows rather than being assigned to any single molecule or specific reaction pathway.

Together with the amorphous features revealed by XRD (Section 3.4), the TG behaviour suggests that solid-state structure and moisture affinity jointly shape the thermal response. Amorphous solids are generally more prone to moisture uptake and may undergo structural relaxation or partial recrystallisation under unfavourable storage conditions [31]. Therefore, for practical handling and downstream processing, low-humidity, sealed, light-protected storage and avoidance of elevated temperatures are advisable to minimise thermal degradation and quality fluctuations. Overall, the TG/DTG results provide direct evidence for maturity-dependent differences in thermal stability and offer actionable guidance for setting processing and storage conditions.

### 3.6 Stability experiment of TFC

As shown in Fig. 3F–H, the TFC of all three flavonoid preparations decreased progressively under natural light during storage, whereas it remained largely stable under sealed, light-protected conditions, with a significant difference between the two treatments. These results indicate that light exposure is a dominant external driver of TFC decay in *A. spinulosa* leaf flavonoids.This behaviour reflects the photosensitivity of flavonoids such as quercetin, where light promotes photo-oxidation and disrupts the conjugated chromophore, leading to reduced UV–Vis absorbance [32]. From a practical standpoint, both the total flavonoid powders and their solutions should therefore be stored and transported under light-protected conditions (e.g., amber containers, aluminium-foil wrapping, or opaque packaging) to minimise photo-induced degradation and reduce batch-to-batch variability.

With respect to temperature (Fig. 3I–K), no significant changes in TFC were observed over the monitoring period when samples were kept sealed and protected from light at either 4 °C or 25 °C. This suggests that, provided samples are light-protected, sealed, and not subjected to extreme pH, temperature within this low-to-moderate range contributes little to the apparent loss of TFC. This finding is operationally relevant for routine handling and short- to mid-term storage, implying that strict refrigeration may not be essential under controlled light and moisture conditions. Nevertheless, kinetic studies have shown that flavonoid degradation rate constants can increase substantially at elevated temperatures [33], and thus high-temperature exposure should still be avoided in processing scenarios or long-term storage.

The pH stability results (Fig. 3L) showed that TFC remained relatively stable across pH 1–9 but declined markedly under stronger alkaline conditions. This trend agrees with prior reports indicating that flavonoids such as quercetin and rutin are prone to deprotonation under alkaline conditions and may subsequently undergo auto-oxidation or structural transformations, resulting in reduced apparent content and/or diminished spectroscopic responses [34]. It should also be noted that colourimetric/UV–Vis assays can be sensitive to ionisation state and absorption behaviour; therefore, the observed decrease under strong alkali may reflect genuine chemical degradation and/or pH-dependent spectral changes. Even so, the overall conclusion that strong alkaline conditions are unfavourable whereas weakly acidic to neutral conditions are comparatively stable aligns with the broader consensus on flavonoid stability [34]. Accordingly, for potential development of extracts or functional formulations, it is advisable to maintain the system within a weakly acidic to neutral pH range and to avoid strong alkaline environments or prolonged alkaline treatment, thereby reducing losses in activity and risks of colour drift.

### 3.7 Targeted flavonoid LC–MS/MS analysis

#### 3.7.1 Data quality control and global features of the *a. Spinulosa* leaf flavonoid profile

**Fig. 4:**
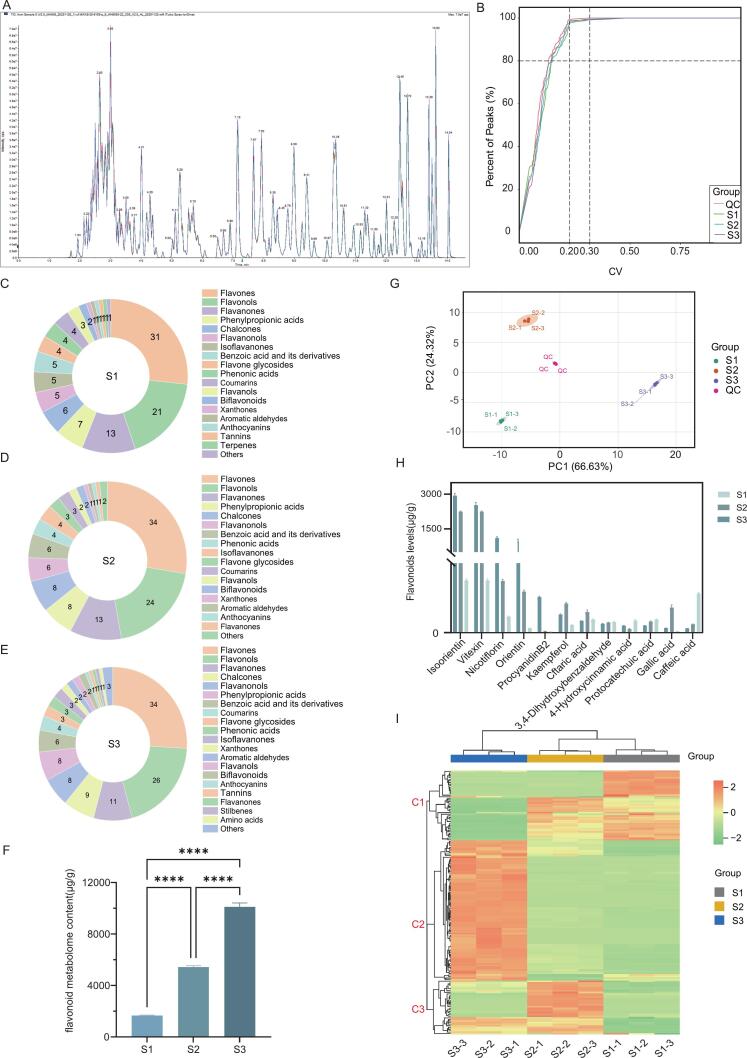
Targeted flavonoid metabolomics of *A. spinulosa* leaves at three maturity stages (S1-S3) based on UPLC-MS/MS. (A) Total ion chromatograms. (B) Cumulative CV distribution of metabolites in QC and biological samples. (C-E) Chemical class composition of annotated metabolites in S1, S2, and S3. (F) Total flavonoid metabolome content in S1-S3. (G) PCA score plot. (H) Concentrations of representative high-abundance metabolites. (I) Hierarchical clustering heatmap of metabolite abundances. Data are presented as mean ± SD (n = 3); ****P < 0.0001.

To delineate the flavonoid landscape of *A. spinulosa* leaves at different maturity grades (S1–S3), we performed targeted qualitative and quantitative analysis using an MRM-based platform supported by a library covering 345 flavonoid standards. The total ion chromatograms (TICs) of the three groups showed highly consistent peak shapes and retention-time distributions (Fig. 4A), indicating stable chromatographic separation and MS acquisition. Data reproducibility was further evaluated using pooled quality-control (QC) samples. As shown in the coefficient of variation (CV) distribution (Fig. 4B), more than 80% of the detected metabolites exhibited CV values below 0.30 in the QC and biological samples, meeting commonly used LC–MS metabolomics criteria for QC filtering and data reliability (CV 20%–30% is typically considered an acceptable threshold) [35]). In addition, PCA revealed tight clustering of QC injections and a clear separation from the biological samples (Fig. 4G, suggesting minimal instrument drift and within-batch fluctuation; therefore, the observed group differences mainly reflect genuine compositional changes across samples rather than analytical artefacts [36]. Importantly, S1–S3 samples were clearly separated along PC1 (explaining 66.63% of the variance), indicating that leaf maturity drives a systematic remodelling of the flavonoid profile rather than sporadic fluctuations in a few compounds.

A total of 166 metabolites were annotated and assigned to 19 classes, including flavones, flavonols, dihydroflavonoids, phenylpropionic acids, chalcones, and dihydroflavonols (Fig. 4C–E). Across all maturity grades, flavones and flavonols predominated in terms of metabolite numbers, and their combined abundance increased with maturity: 31 + 21 (52) in S1, 34 + 24 (58) in S2, and 34 + 26 (60) in S3. This pattern is consistent with the physiological roles of foliar flavonoids in photoprotection and redox buffering: as leaves mature and experience greater cumulative light exposure, plants often increase flux through the phenylpropanoid pathway towards flavonoid biosynthesis to enhance ROS scavenging capacity and tolerance to photo-oxidative stress [37]. Accordingly, the “expansion” and “accumulation” of the flavonoid repertoire in S3 not only implies higher extractability, but also points to a maturity-stage chemical strategy that supports adaptation to environmental challenges (e.g., high irradiance and temperature fluctuations) [38].

Quantitative results further corroborated this maturity-associated accumulation trend. When the summed abundance of the 166 metabolites was used as a proxy for the “total flavonoid metabolome”, S3 exhibited significantly higher levels than S1 and S2 (Fig. 4F). At the individual-compound level, isoorientin, vitexin, nicotiflorin, and orientin were the four most abundant constituents across all samples (ranked by mean abundance across S1–S3), and their concentrations were of a markedly higher order of magnitude than most other flavonoids (Fig. 4H). Notably, isoorientin, vitexin, and orientin are C-glycosyl flavones. In many plant leaves, C-glycosides show pronounced tissue specificity and a tendency to accumulate, and the C–C glycosidic linkage is often associated with enhanced chemical stability and improved tolerance to processing compared with O-glycosides [39]. This is consistent with their prominent enrichment in S3. In S3, these four high-abundance flavonoids reached 2932.457 ± 107.644, 2522.770 ± 119.030, 1102.463 ± 53.717, and 991.557 ± 61.651 μg/g, respectively, showing a clear accumulation phenotype in mature leaves. Conversely, a small subset of phenolic acids/phenylpropanoid-related compounds showed an opposite pattern. For example, caffeic acid reached 280.943 ± 9.288 μg/g in S1 but decreased to 60.229 ± 2.303 μg/g and 30.696 ± 0.727 μg/g in S2 and S3, respectively. Given that hydroxycinnamic acids such as caffeic acid occupy key nodes in the phenylpropanoid network and can act as precursors and/or companion metabolites for downstream phenolics (including flavonoids), its relative enrichment in young leaves may suggest that S1 favours a larger “precursor pool” or basal phenolic defence, whereas S3 shifts towards deposition of more complex flavonoid glycosides as end products—reflecting a development-associated redistribution of metabolic flux [35].

To visualise coordinated abundance changes across maturity at the global level, we performed bidirectional hierarchical clustering of the auto-scaled 166 metabolites using Euclidean distance and Ward’s linkage (Fig. 4I). S1, S2, and S3 formed three distinct branches along the horizontal axis, and biological replicates clustered tightly within each group, further supporting the robustness and biological consistency of the maturity-associated differences. The heatmap broadly revealed three metabolite modules. Cluster C1 comprised metabolites relatively enriched in S1 (e.g., protocatechuic acid, 96.210 ± 5.318 μg/g; 4-hydroxycinnamic acid, 86.626 ± 7.121 μg/g) that gradually declined with maturity; some compounds were detected only in S1 (e.g., 3-methoxytangeretin and quercetin 3-sambubioside). In contrast, cluster C2 displayed an accumulation pattern with maturity, reaching high levels in S3 (e.g., apigenin-4′-O-glucoside, 119.845 ± 3.500 μg/g; nicotiflorin, 1102.463 ± 53.717 μg/g), consistent with enhanced deposition of flavonoid end products in mature leaves [40]. Cluster C3 showed S3-specific upregulation (e.g., nepitrin, 52.679 ± 2.867 μg/g), suggesting that, beyond broadly accumulating flavonoids, certain secondary-metabolic branches may be selectively activated or intensified at late maturity (Details of Cluster 1–3 are provided in Supplementary material 2, Table S4). Overall, the clustering pattern mirrored the PCA separation and supports the conclusion that leaf maturity is a primary driver of flavonoid-profile remodelling in *A. spinulosa*. The results also imply practical differentiation in raw-material selection: S3 appears more favourable when C-glycosyl flavones are desired, whereas S1 may be more representative for phenolic acids or early-stage marker metabolites.

#### 3.7.2 Differential flavonoid analysis

To further delineate maturity-dependent divergence in the flavonoid profiles of *A. spinulosa* leaves (S1–S3), an orthogonal partial least squares–discriminant analysis (OPLS-DA) model was constructed based on the targeted quantitative dataset. OPLS-DA separates predictive variation related to class discrimination from orthogonal (class-unrelated) variation, thereby improving interpretability and facilitating the identification of discriminant metabolites [41]. In metabolomics, OPLS-DA is typically used in conjunction with cross-validation and permutation testing to mitigate the risk of overfitting, particularly in settings with high-dimensional variables and relatively limited sample sizes [42].

**Fig. 5:**
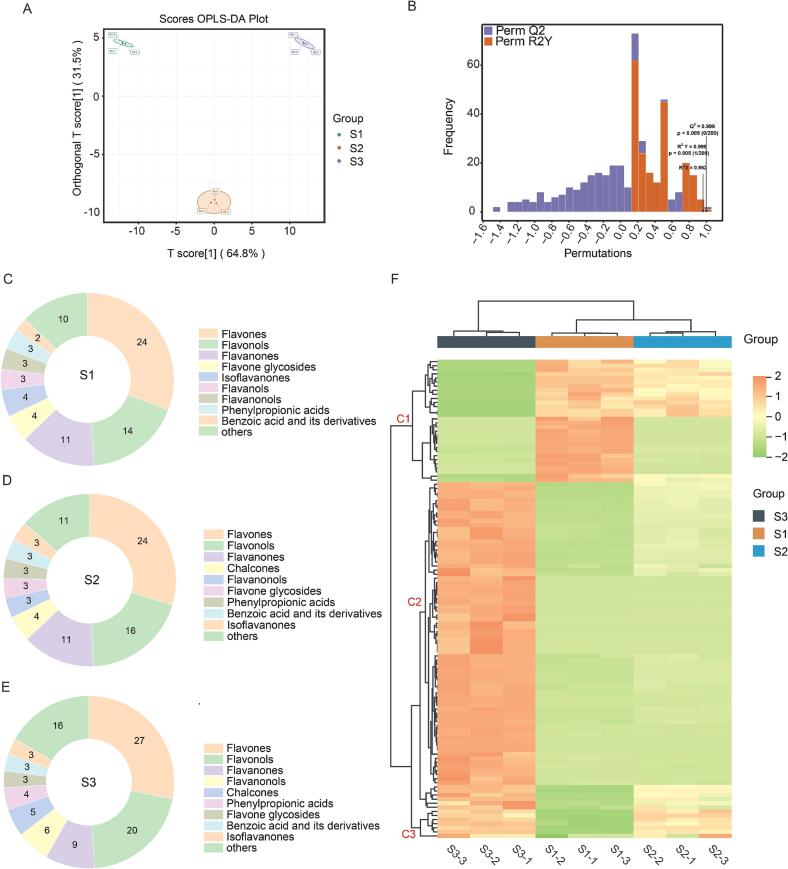
OPLS-DA and clustering analysis of differential flavonoid metabolites among the three maturity stages of *A. spinulosa* leaves. (A) OPLS-DA score plot. (B) Permutation test of model robustness. (C-E) Chemical class distribution of differential metabolites in S1, S2, and S3. (F) Hierarchical clustering heatmap of differential metabolites.

As shown in Fig. 5A, S1, S2, and S3 samples were clearly separated along the predictive component, with tight clustering among biological replicates, indicating systematic maturity-associated differences in the flavonoid metabolome. In this model, t[1] (64.8%) captured the major between-group discriminatory information, whereas the orthogonal component (31.5%) reflected within-group variability, suggesting that the observed separation was not driven by a few outliers. Model statistics (R^2^X = 0.962, R^2^Y = 0.999, Q^2^ = 0.999) indicated excellent goodness-of-fit and strong cross-validated predictive performance. Moreover, permutation testing (Fig. 5B) yielded substantially lower model statistics for randomly permuted class labels than for the original model, supporting that the discrimination was not attributable to chance fitting and that the model was robust [43].

Using a VIP > 1 threshold, 119 differential metabolites were identified (Table S5 in Supplementary material 2). In terms of chemical subclass composition (Fig. 5C–E), these differential metabolites predominantly fell into 10 categories, with flavones and flavonols accounting for the largest proportions, indicating that maturity-related divergence in *A. spinulosa* leaves is mainly concentrated within these two structural types. Notably, flavones remained the largest category across stages (24, 24, and 27 compounds in S1, S2, and S3, respectively), with a slight increase at S3. In contrast, flavonols increased progressively with maturity (14 in S1, 16 in S2, and 20 in S3), implying that hydroxylation of the flavonoid scaffold and subsequent modifications (e.g., glycosylation and/or acylation) become increasingly prominent as leaves mature. This trend aligns with the widely recognised roles of flavonols in foliar photoprotection and oxidative-stress modulation [44]. By comparison, subclasses such as flavanols, flavanonols, and phenylpropionic acids showed relatively stable representation across groups, suggesting that they contribute less to the primary discriminatory signal between maturity stages.

Bidirectional hierarchical clustering (Fig. 5F) provided an independent global confirmation of these patterns. Along the horizontal axis, S1–S3 formed three distinct branches, and biological replicates clustered closely within each group, demonstrating good reproducibility and consistency with the OPLS-DA separation. Along the vertical axis, several metabolite modules were resolved (labelled C1–C3, details of C1–C3 are provided in Supplementary material 2, Table S6). The C1 module was relatively enriched in S1, representing an “early-accumulation” pattern. This module included, for example, caffeic acid and 4-hydroxycinnamic acid (phenylpropionic acids), protocatechuic acid and salicylic acid (benzoic acid derivatives), and biflavonoids such as sciadopitysin and ginkgetin. Such compounds are frequently associated with early-stage chemical defence and stress acclimation during the vulnerable juvenile window, when tissues may be particularly sensitive to UV exposure and pathogen pressure [45].

In contrast, the C3 module showed higher abundance in S3 and reflected a “late-accumulation” pattern. As leaves mature, metabolic flux within the phenylpropanoid network may shift from relatively simple, upstream phenolics towards downstream flavonoid glycosides, consistent with maturation-driven reinforcement of photoprotective and redox-buffering capacity. Accordingly, compared with S1, S3 leaves displayed marked increases in apigenin-4′-O-glucoside, luteolin-3′,7-di-O-glucoside, isoorientin, and sieboldin. The enrichment of both O-glycosides and C-glycosides supports the notion that soluble flavonoid glycosides represent an important chemical strategy for mature leaves to maintain photoprotection and redox homeostasis [46]. The C2 module likely captured transitional or stage-dependent fluctuations, featuring metabolites such as sophoricoside, nicotiflorin, and isoorientin that increased progressively across S1–S3. This pattern is compatible with a gradual upregulation of glycosylated flavones and flavonols during maturation, potentially reflecting enhanced investment in protective secondary metabolism under increasing cumulative environmental exposure [44].

Collectively, the OPLS-DA and clustering analyses indicate that maturity-associated divergence in *A. spinulosa* leaves involves both (i) compounds that accumulate continuously with development and (ii) early-stage dominant metabolites that decline as leaves mature [47]. This structured remodelling provides a coherent chemical basis for subsequent prioritisation of maturity-specific marker compounds and for linking compositional shifts to downstream functional readouts.

### 3.8 Bioactivities of total flavonoids from *a. Spinulosa* leaves in LPS-stimulated RAW 264.7 macrophages

**Fig. 6:**
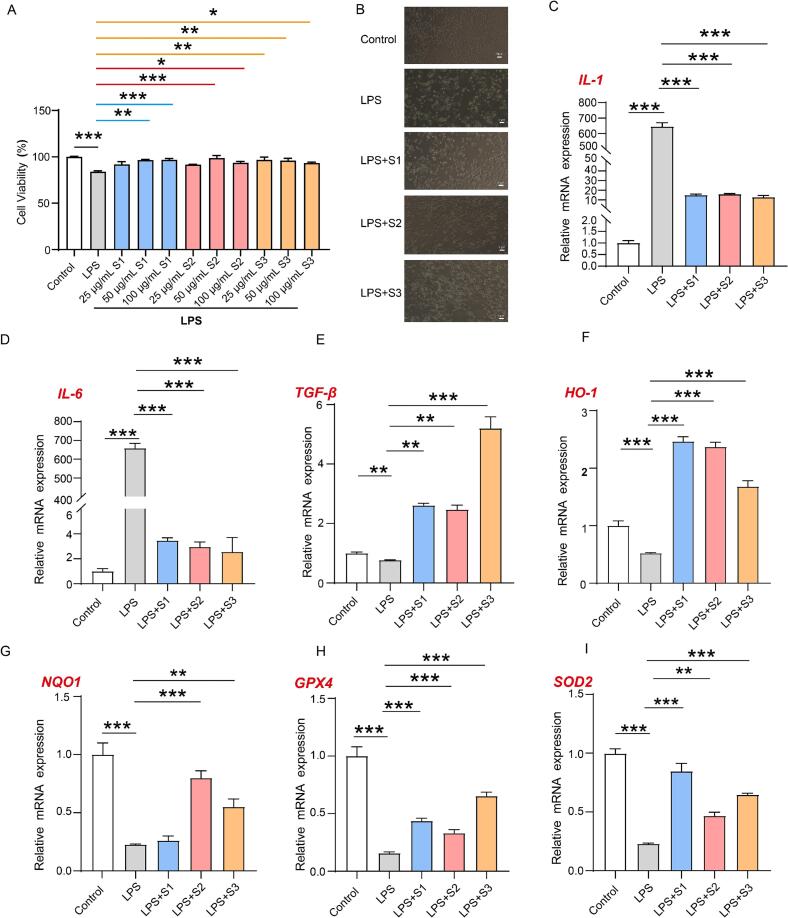
Effects of *A. spinulosa* flavonoid extracts on cell viability, morphology, and gene expression in LPS-stimulated RAW 264.7 macrophages. (A) Cell viability after treatment with flavonoid extracts at 25, 50, and 100 μg/mL. (B) Representative cell images of the control, LPS, and LPS + S1-S3 groups. (C-I) Relative mRNA expression of IL-1, IL-6, TGF-β, HO-1, NQO1, GPX4, and SOD2. Data are presented as mean ± SD (n = 3). Statistical significance was determined by one-way ANOVA followed by Tukey’s test (*P < 0.05, **P < 0.01, ***P < 0.001).

To evaluate the anti-inflammatory potential and the cytoprotective responses associated with oxidative stress, RAW 264.7 macrophages were exposed to *A. spinulosa* total flavonoid extracts prepared from leaves at three maturity stages (S1–S3), and an LPS-stimulated inflammatory model was used for readouts. First, cell viability was assessed by CCK-8 across 25, 50, and 100 μg/mL (Fig. 6A). Within this range, the extracts did not cause overt cytotoxicity, with viability remaining between 81% and 100%. Therefore, 50 μg/mL was selected for subsequent experiments to ensure biological responsiveness while avoiding confounding toxicity.

Morphological changes induced by LPS are shown in Fig. 6B, consistent with successful inflammatory stimulation. At the transcriptional level, LPS markedly increased the mRNA expression of the pro-inflammatory cytokines IL-1 and IL-6 relative to the untreated control (Fig. 6C–D, P < 0.001), confirming effective activation of inflammatory signalling. Co-treatment with total flavonoid extracts (S1–S3) significantly attenuated LPS-induced cytokine upregulation (Fig. 6C–D, P < 0.001). In particular, the S3 extract produced a stronger reduction in these transcripts under the present conditions (IL-1 and IL-6 decreased to 1/50 and 1/257 of the LPS group, respectively; Fig. 6C–D), suggesting that maturity-associated compositional differences may influence the magnitude of anti-inflammatory transcriptional responses.

In addition to classical pro-inflammatory markers, TGF-β was analysed as an immune-regulatory cytokine. Notably, TGF-β is pleiotropic and its biological directionality depends on cellular context and stimulation background; thus, in this model it is more appropriate to interpret TGF-β as an immune-regulatory signal rather than a simple “anti-inflammatory factor” [48]. As shown in Fig. 6E, TGF-β mRNA was significantly elevated in the S3 co-treatment group (approximately 6.8-fold versus LPS), implying that the extract may modulate immune-regulatory programmes during LPS-triggered activation.

Because LPS-driven inflammation is frequently accompanied by disturbed redox homeostasis, we further examined transcriptional responses of representative antioxidant and cytoprotective genes. Compared with the untreated control, LPS exposure significantly reduced HO-1, NQO1, GPX4, and SOD2 expression (Fig. 6F–I, P < 0.001), consistent with an impaired cellular defence profile under inflammatory stress [49]. Co-treatment with the S1–S3 total flavonoid extracts significantly restored the expression of these genes (Fig. 6F–I, P < 0.001), indicating that the extracts can counteract LPS-associated suppression of redox-defence transcription. Differences in response magnitude were also observed among extracts; for instance, HO-1 induction in the S1 group was numerically higher (1.5-fold versus LPS; Fig. 6F), although this should be interpreted cautiously as a transcriptional endpoint rather than direct evidence of enzymatic activity or ROS-scavenging capacity.

Taken together, the total flavonoids from *A. spinulosa* leaves substantially mitigated LPS-induced inflammatory gene expression (IL-1β and IL-6) and promoted a more favourable cytoprotective transcriptional profile (HO-1, NQO1, GPX4, and SOD2) in RAW 264.7 macrophages.

### 3.9 Molecular docking analysis

**Fig. 7:**
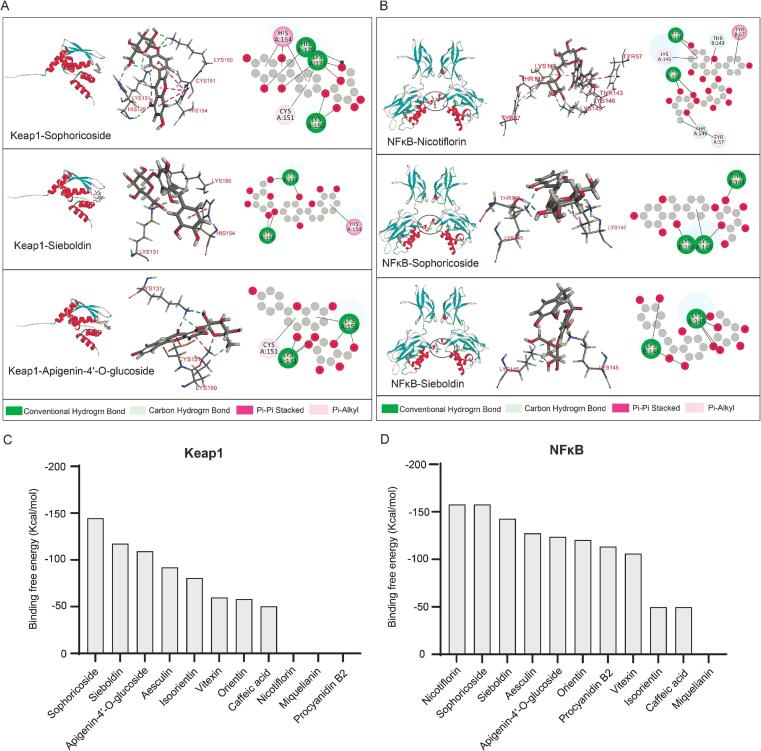
Molecular docking of representative flavonoids from *A. spinulosa* leaves with Keap1 and NF-κB. (A) Docking interactions of Sophoricoside, Sieboldin, and Apigenin-4′-O-glucoside with Keap1. (B) Docking interactions of Nicotiflorin, Sophoricoside, and Sieboldin with NF-κB. (C) Binding free energies for docking with Keap1. (D) Binding free energies for docking with NF-κB.

To further probe the potential bioactivities of *A. spinulosa* leaf flavonoids, the 12 most abundant metabolites (Fig. 4H; all also identified as differential metabolites) were prioritised for molecular docking against two functionally relevant targets, the antioxidant-related sensor Keap1 (Fig. 7A) and the inflammatory transcription factor NF-κB (Fig. 7B) [50]. Docking scores were used to rank candidates and to compare their predicted binding propensities within the corresponding ligand-binding cavities, thereby providing a structure-informed rationale for subsequent monomer selection and functional validation.

As shown in Fig. 7A, the 12 metabolites displayed markedly different predicted affinities towards Keap1, indicating heterogeneous potential in modulating Keap1-associated redox regulation. Among them, Sophoricoside, Sieboldin and Apigenin-4′-*O*-glucoside achieved the top three docking scores (−144.63, −117.46 and − 109.31 kcal/mol, respectively). Visual inspection of the representative poses suggested that these ligands could be accommodated within the predicted Keap1 cavity via multiple noncovalent interactions (for example, hydrogen bonding and hydrophobic contacts), supporting their prioritisation as candidate Keap1-binding flavonoids. Notably, docking provides a static, structure-based estimate of binding plausibility rather than direct evidence of pathway activation; therefore, these results are interpreted primarily as a prioritisation tool rather than a mechanistic conclusion.

For NF-κB (Fig. 7B), Nicotiflorin, Sophoricoside and Sieboldin were ranked as the top binders, with docking scores of − 157.75, −157.75 and − 142.68 kcal/mol, respectively, suggesting that these flavonoids may possess higher binding propensity towards the selected NF-κB cavity. The predicted binding conformations implied contributions from noncovalent interactions (including polar contacts and aromatic interactions) stabilising ligand occupancy within the pocket, which is consistent with their prioritisation for downstream anti-inflammatory validation.

Importantly, Sophoricoside and Sieboldin emerged as shared high-priority candidates for both Keap1 and NF-κB, highlighting them as potentially multifunctional flavonoids in *A. spinulosa* leaves. It has been evidenced that Sophoricoside (genistein-4′-*O*-glucoside) has been reported to alleviate autoimmune hepatitis with concurrent attenuation of oxidative stress and inflammatory responses, involving regulation of AMPK/Nrf2 and NF-κB-associated signalling [51]. In addition, Sophoricoside was shown to reduce inflammatory mediator production in LPS-challenged models, supporting its anti-inflammatory potential in immune contexts [52]. For Sieboldin (3-hydroxyphlorizin), comparative evaluations of dihydrochalcones have reported strong radical-scavenging capacity for 3-hydroxyphlorizin, consistent with its prioritisation as an antioxidant-related candidate [53].

### 3.10 Bioactivity validation of representative flavonoid monomers

Guided by the high-abundance signals in the targeted flavonoid dataset (Fig. 4H) and the docking-based prioritisation (Fig. 7), six representative monomers were selected for cell-level validation: vitexin (1), isoorientin (2), orientin (3), kaempferol-3-*O*-rutinoside (nicotiflorin) (4), sophoricoside (5), and sieboldin (6). Among them, sophoricoside and sieboldin were consistently ranked as high-priority ligands against both Keap1 and NF-κB in silico, whereas vitexin, isoorientin, orientin and nicotiflorin represented the major constituents detected at high abundance in the extracts. This combined “abundance plus docking priority” strategy aimed to balance chemical representativeness with mechanistic plausibility when interrogating inflammation- and redox-defence–related transcriptional outputs under an LPS challenge.

**Fig. 8:**
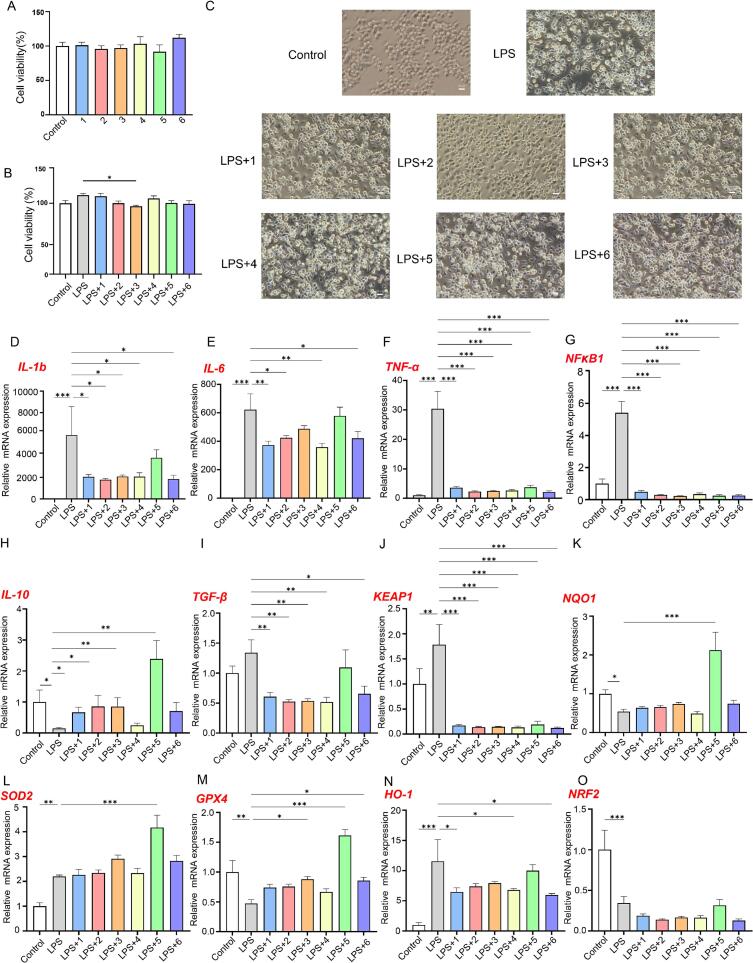
Effects of representative flavonoid metabolites from *A. spinulosa* leaves on cell viability, morphology, and gene expression in LPS-stimulated RAW 264.7 macrophages. (A, B) Cell viability after treatment with flavonoid metabolites (1–6) at 50 μg/mL. (C) Representative cell images of the control, LPS, and LPS + 1–6 groups. (D-O) Relative mRNA expression of IL-1β, IL-6, TGF-α, NFKB1, IL-10, TGF-β, KEAP1, NQO1, SOD2, GPX4, HO-1, and NRF2. Data are presented as mean ± SD (n = 3). Statistical significance was determined by one-way ANOVA followed by Tukey’s test (*P < 0.05, **P < 0.01, ***P < 0.001)..

At 10 μM, none of the six monomers produced appreciable cytotoxicity in RAW 264.7 macrophages after 24 h, with cell viability remaining close to the untreated control (Fig. 8A). Under LPS co-treatment, viability also stayed high across groups (generally above ∼95%; Fig. 8B), supporting the view that subsequent RT–qPCR differences largely reflected transcriptional regulation rather than confounding effects from extensive cell loss. Morphologically, LPS induced a typical activation/stress-like phenotype (e.g., more rounded cells, altered adherence and increased clustering; Fig. 8C), while co-incubation with individual flavonoids alleviated these changes to varying degrees (Fig. 8C), consistent with the directionality of the gene-expression readouts.

RT–qPCR analysis showed that LPS robustly increased the mRNA levels of *IL-1b*, *IL-6*, *TNF-α* and *NFKB1* compared with untreated cells (P < 0.001; Fig. 8D–G), in line with canonical TLR4-driven inflammatory activation in macrophages (Zeshan [54]). Co-treatment with each flavonoid attenuated these pro-inflammatory transcriptional signals, although the magnitude varied by compound. The dampening was particularly evident for *TNF-α* and *NFKB1*: relative to the LPS-only group, their expression decreased to approximately 1/14–1/8 and 1/24–1/11, respectively (Fig. 8F–G). Taken together, these data suggest that the selected monomers can restrain LPS-evoked inflammatory gene output, plausibly through interference with NF-κB-centred transcriptional activation [55], [56].

For immune-modulatory readouts, *IL-10* is commonly associated with inflammation resolution and negative immune regulation [57]. By contrast, *TGF-β* encodes a pleiotropic cytokine whose biological directionality depends strongly on cell type and context, spanning immune suppression, tissue repair and pro-fibrotic programmes; therefore, within the RAW 264.7–LPS setting it is more appropriate to discuss *TGF-β* as an immune-modulatory signal rather than assigning it a single “anti-inflammatory” label. In this dataset, several monomers promoted recovery or upregulation of *IL-10* under LPS exposure (Fig. 8H), with sophoricoside (5) showing a more evident effect, while *TGF-β* responses differed among compounds (Fig. 8I), indicating that these flavonoids may not share an identical immunomodulatory profile.

LPS stimulation also coincided with altered redox-defence–related transcription. A tendency towards increased *KEAP1* mRNA was observed in the LPS group, whereas all six monomers significantly reduced *KEAP1* transcripts under co-treatment (Fig. 8J). In parallel, expression of key cytoprotective genes linked to redox defence, including *NQO1*, *SOD2* and *GPX4*, was increased to varying extents (Fig. 8K–M), suggesting that these monomers can reinforce cellular defence-associated transcriptional programmes in an inflammatory background [58]. Notably, sophoricoside (5) elicited a comparatively larger induction of *NQO1*, *SOD2* and *GPX4* (Fig. 8K–M; fold changes shown in the bar plots) alongside reduced *KEAP1* (Fig. 8J), which aligns directionally with its high docking priority (ranked first for Keap1 and second for NF-κB; Fig. 7C–D). While docking alone only indicates structural binding plausibility, the concordant transcriptional response provides functional support that sophoricoside is a particularly promising candidate for deeper mechanistic validation.

Although the six representative flavonoids identified in *A. spinulosa* exhibited marked anti-inflammatory and antioxidant activities in LPS-stimulated RAW 264.7 macrophages, their in vivo efficacy after oral administration will depend on gastrointestinal stability, intestinal absorption, first-pass metabolism, and microbial biotransformation. Available pharmacokinetic evidence indicates marked compound-dependent differences. Vitexin shows low oral bioavailability in rats, with an absolute oral bioavailability of 4.91 ± 0.76% [59], and undergoes substantial first-pass loss, especially in the intestine, with reported hepatic, gastric, and intestinal first-pass effects of 5.2%, 31.3%, and 94.1%, respectively [60]. Isoorientin likewise exhibits low oral bioavailability in rats, with a reported value of 8.98 ± 1.07% [61], although orientin and isoorientin have been shown to cross Caco-2 monolayers mainly by passive diffusion and are considered relatively permeable [62]. For kaempferol-3-O-rutinoside (nicotiflorin), the reported oral bioavailability was 2.00%, indicating limited systemic exposure after oral administration [63]. Sophoricoside was reported to be rapidly absorbed and eliminated in rats, and its biological effects may also involve conversion to genistein, which was simultaneously detected as a circulating metabolite [64]. For sieboldin, direct pharmacokinetic or absolute oral bioavailability data are still unavailable. However, sieboldin is an O-glycosylated dihydrochalcone, namely 3-hydroxyphloretin 4′-O-glucoside, and dihydrochalcones are generally considered to be limited by low solubility and bioavailability [65]. In addition, structurally related apple dihydrochalcones such as phloridzin and phloretin have been reported to show poor absorption or low oral bioavailability, suggesting that the in vivo exposure of sieboldin may also be limited and should be verified in future studies [66] Therefore, the anti-inflammatory potential of the *A. spinulosa* flavonoid fraction may depend not only on the native compounds themselves but also on their metabolic conversion in vivo. Nevertheless, the present study did not assess bioaccessibility, pharmacokinetics, or tissue exposure, and further in vivo studies are required to clarify the translational relevance of these flavonoids.

Finally, interpretation of “antioxidant” readouts should consider model specificity. The RAW 264.7–LPS system is a classical inflammation model rather than a dedicated oxidative-stress model; therefore, certain defence-gene behaviours may not fully mirror those observed under oxidant-centred challenges (e.g., H_2_O_2_, *tert*-butyl hydroperoxide, AAPH/ABAP, or menadione) [67]. This context may partly explain why some genes sometimes deviate from conventional expectations, and follow-up experiments using oxidative-stress–focused paradigms would help to refine the redox-related mechanistic conclusions.

## 4 Conclusion

In this work, *A. spinulosa* leaves across a maturity gradient (S1–S3) were used to establish an integrated workflow that links extraction engineering, chemical interpretation and bioactivity validation. A Box–Behnken RSM model defined a robust UAE window for efficiently enriching total flavonoids, while complementary solid-state and stability characterisation highlighted practical handling requirements, particularly light protection and avoidance of strong alkaline conditions. Targeted LC–MS/MS profiling demonstrated that leaf maturity is a key determinant shaping the flavonoid landscape, with a clear shift from early-stage phenolic acids towards the accumulation of flavone/flavonol glycosides in more mature leaves. Functionally, both purified extracts and selected representative monomers, namely vitexin, isoorientin, orientin, kaempferol-3-O-rutinoside (nicotiflorin), sophoricoside and sieboldin, attenuated LPS-triggered inflammatory transcriptional outputs in RAW 264.7 macrophages and concurrently modulated defence-related gene responses under the same inflammatory background. By integrating abundance information with docking-based prioritisation, the study further narrows down candidate constituents for future mechanistic work and standardisation efforts, thereby providing a practical basis for identifying and developing bioactive constituents from *A. spinulosa* leaves.

Nevertheless, further in vivo studies are needed to clarify the bioavailability, metabolic fate, and systemic effects of these flavonoids after oral administration. In addition, protein-level validation of the key inflammatory and defence-related targets will be necessary to further substantiate the physiological relevance and mechanistic basis of the present findings.

## CRediT authorship contribution statement

**Xiong Huang:** Writing – review & editing, Visualization, Funding acquisition, Data curation. **Yingying He:** Writing – review & editing, Formal analysis, Data curation. **Wenjing Miu:** Investigation. **Yu Sui:** Investigation. **Pengpeng Gong:** Resources. **Ruixue Yuan:** Resources. **Xuelian Tang:** Methodology. **Chen Liu:** Supervision, Funding acquisition.

## Funding

This work was supported by the 10.13039/501100001809National Natural Science Foundation of China [Grant No. 32301612]; Natural Science Foundation of Sichuan Province [Grant No. 2025ZNSFSC0994]; China Postdoctoral Science Foundation [Grant No. 2023M742510]; Lhasa Science and Technology Project: Development of Low-Fluoride High-Quality Tibetan Tea Products [Grant No. LSKJ202534].

## Declaration of competing interest

The authors declare that they have no known competing financial interests or personal relationships that could have appeared to influence the work reported in this paper.
