# Supplementary material for: Ultrasound extraction and bioactive flavonoid profiling of Alsophila spinulosa leaves across maturity stages
Source: Ultrason Sonochem. 2026 Apr 13;129:107847. doi: 10.1016/j.ultsonch.2026.107847 (PMC13126033; doi:10.1016/j.ultsonch.2026.107847)
Supplement: Supplementary Data 1 [file mmc1.pdf]

Ultrasound Extraction and Bioactive Flavonoid Profiling of *Alsophila spinulosa* Leaves Across Maturity Stages

Xiong Huang[1#], Yingying He[1#], Wenjing Miu[1], Yu Sui[1], Pengpeng Gong[1], Ruixue Yuan[1], Xuelian Tang[1], Chen Liu[2]*

[1]College of Forestry, Forest Ecology and Conservation in the Upper Reaches of the Yangtze River Key Laboratory of Sichuan Province, Sichuan Agricultural University, Chengdu, 611130, Sichuan, P. R. China

[2]College of Horticulture, Sichuan Agricultural University, Chengdu, 611130, Sichuan, P. R. China

[*]Corresponding author: Chen Liu (liuchen@sicau.edu.cn).

[#]These authors contribute equally to this work.

Table S1. Experimental design of single factor experiment

Factors	B<br>Ultrasound power（W	D<br>Ethanol content（%）	C<br>Liquid-soild ratio（g/mL）	A<br>Ultrasound time（min）
A<br>Ultrasound time（min）	300	40	30	20,40,60,80,<br>100,120,140
B<br>Ultrasound power（W）	100,200,300,<br>400,500	40	30	30
C<br>Liquid-soild ratio（g/mL）	300	40	1:20,40,60,8<br>,100,120	30
D<br>Ethanol content（%）	300	20,40,60,80,<br>100	30	30

Table S2. Factors and levels of Box-Behnken experiment

Factor level	Factors			
	A<br>Ultrasound time<br>（min）	B<br>Ultrasound power<br>（W）	C<br>Liquid-soild ratio<br>（g/mL）	D<br>Ethanol content<br>（%）
-1	20	100	20	20
0	60	300	60	50
1	100	500	100	80

Table S3. Primer sequences used for real-time quantitative PCR

Genes	Sequence
GAPDH	F:5′-AGGGCATCTTGGGCTACAC-3′
	R:5′-TGGTCCAGGGTTTCTTACTCC-3′
HO-1	F: 5′-GGTACACATCCAAGCCGAGA-3′
	R:5′-GGTACAAGGAAGCCATCACC-3′
NQO1	F:5′-GACATCATTCAACTACGCCAT-3′
	R:5′-TTCCAGCTTCTTGTGTTCGG-3′
GPX4	F:5′-CTCAAGTACGTCCGACCTGG-3′
	R:5′-TAAAGAGCGGGTGAGCCTTC-3′
SOD2	F:5′-CGTGAACAACCTCAACGCC-3′
	R:5′-GTCACGCTTGATAGCCTCCA-3′
IL-1	F:5′-GCTTCCTTGTGCAAGTGTCTGA-3′
	R:5′-TCAAAAGGTGGCATTTCACAGT-3′
IL-1b	F:5′-GCAACTGTTCCTGAACTCAACT-3′
	R:5′-ATCTTTTGGGGTCCGTCAACT-3′
IL-6	F:5′-AAAATTTCCTCTGGTCTTCTGGAGT-3′
	R:5′-TTCTGTGACTCCAGCTTATCTCTTG-3′
TGF-β	F:5′-ATTAAAATCAAGTGTGGAGCAAC-3′
	R:5′-GAAAGCCCTGTATTCCGTCT-3′
TNF-α	F:ATGGCCTCCCTCTCATCAGT
	R:TTTGCTACGACGTGGGCTAC
IL-10	F:5′-GCTCTTACTGACTGGCATGAG-3′
	R:5′-CGCAGCTCTAGGAGCATGTG-3′
NFKB1	F:5′-GGAGGCATGTTCGGTAGTGG-3′
	R:5′-CCCTGCGTTGGATTTCGTG-3′
NRF2	F:5′-CTGAACTCCTGGACGGGACTA-3′
	R:5′-CGGTGGGTCTCCGTAAATGG-3′
KEAP1	F:5′-TGCCCCTGTGGTCAAAGTG-3′
	R:5′-GGTTCGGTTACCGTCCTGC-3′

Table S4. Single-factor screening experiments for factor range determination.

Factors	Range	Tested Levels (n=3)	Optimal Level Observed
A<br>Ultrasound time<br>（min）	20-120	20,30,40,60,80,100,120	60
B<br>Ultrasound power<br>（W）	0-500	0,100,200,300,400,500	300
C<br>Liquid-soild ratio<br>（g/mL）	1:20-1:120	1:20,30,60,80,100,120	60
D<br>Ethanol content<br>（%）	20-80	20,40,50,60,80	50

Table S5. Analysis of variance (ANOVA) for the Box–Behnken response surface quadratic model describing total flavonoid content (TFC) during ultrasound-assisted extraction (UAE) of *A. spinulosa* leaves.

source	Sum of squares	Df	Mean squaer	F-value	P-value	
Model	1125.65	14	80.4	28.39	<0.0001	significant
A Ultrasound time	8.11	1	8.11	2.87	0.1127	
B Ultrasound power	28.32	1	28.32	10	0.0069	
C Liquid-soild ratio	49.48	1	49.48	17.47	0.0009	
D Ethanol content	111.63	1	111.63	39.42	<0.0001	
AB	14.82	1	14.82	5.23	0.0382	
AC	13.81	1	13.81	4.88	0.0444	
AD	0	1	0	0	1	
BC	0.1469	1	0.1469	0.0519	0.8231	
BD	1.1	1	1.1	0.3894	0.5427	
CD	21.62	1	21.62	7.64	0.0152	
A^2	110.71	1	110.71	39.1	<0.0001	
B^2	0.6596	1	0.6596	0.2329	0.6368	
C^2	109.6	1	109.6	38.71	<0.0001	
D^2	790.54	1	790.54	279.18	<0.0001	
Residual	39.64	14	2.83			
Lack of Fit	35.93	10	3.59	3.87	0.102	not significant
Pure Error	3.71	4	0.928			
Cor Total	1165.29	28				

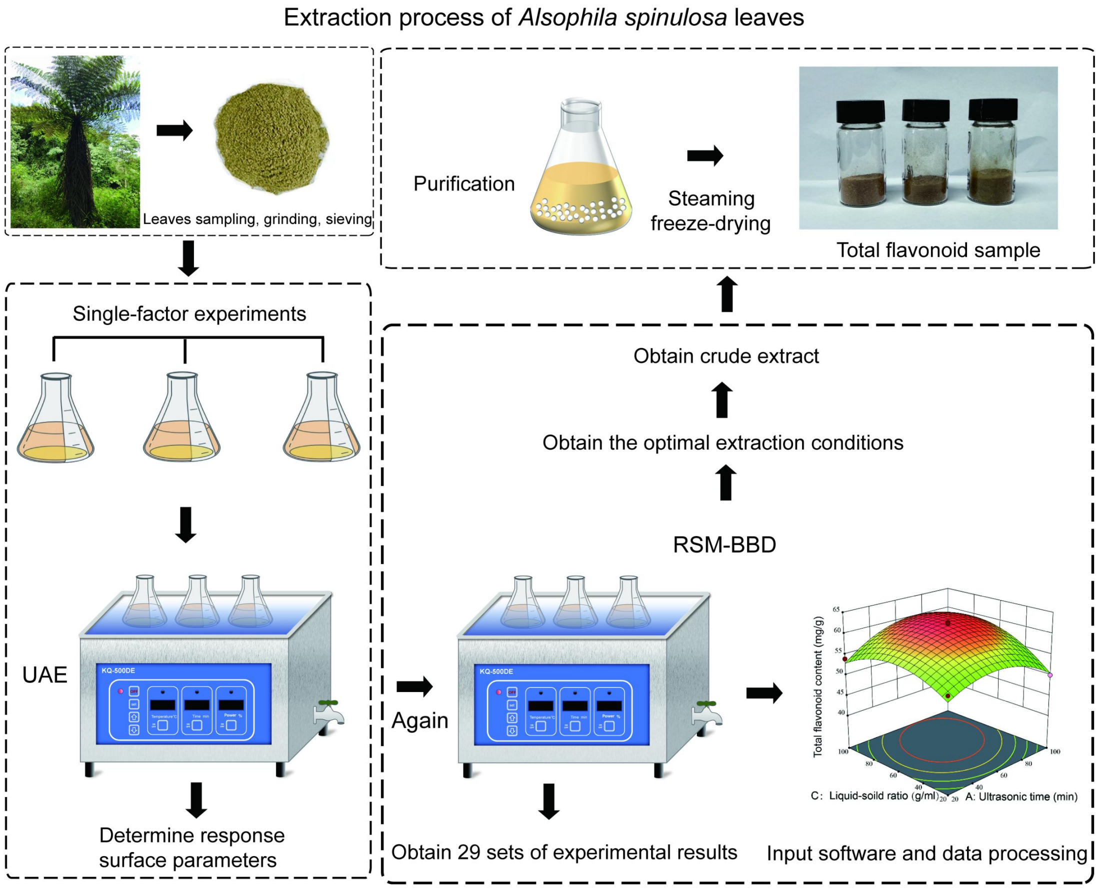

Figure S1. Schematic diagram of the extraction, optimisation, and purification workflow for flavonoids from *A. spinulosa* leaves.

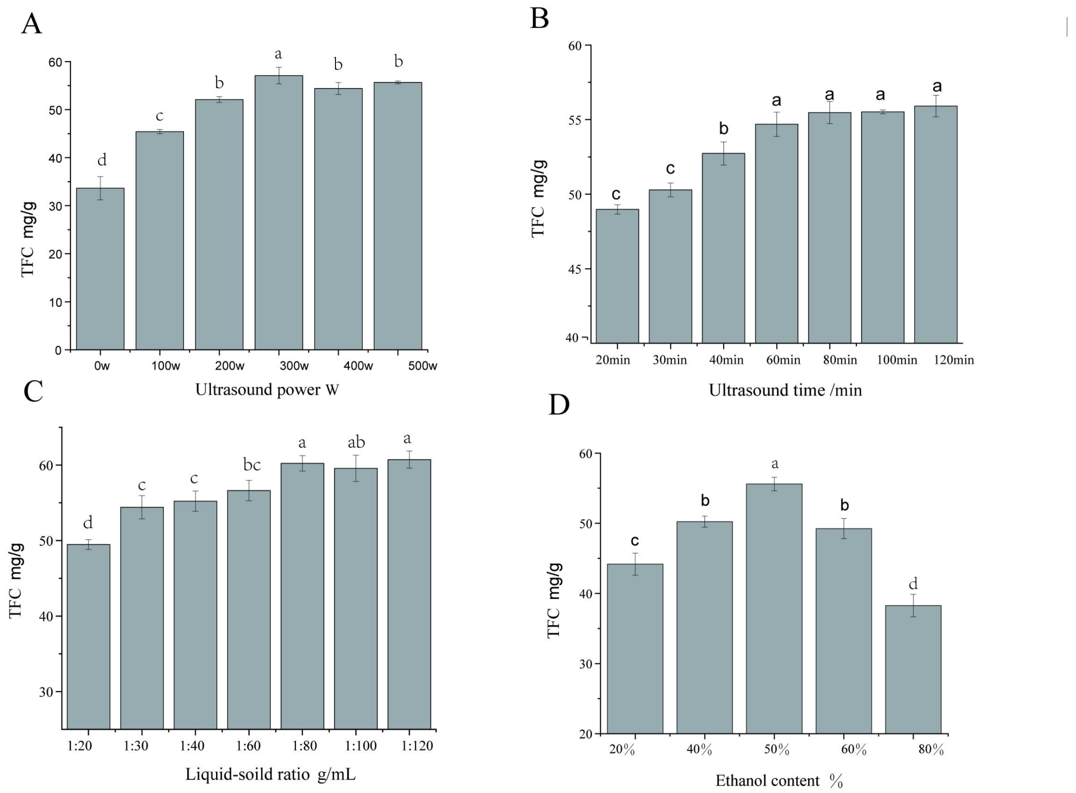

Figure S2. The results of single-factor experiment
